# Chemogenetic modulation of the rat locus coeruleus alters hippocampal noradrenaline release and modulates perforant path-evoked responses

**DOI:** 10.3389/fnins.2025.1544830

**Published:** 2025-02-19

**Authors:** Sielke Caestecker, Emma Lescrauwaet, Kristl Vonck, Mathieu Sprengers, Evelien Carrette, Paul Boon, Lars Emil Larsen, Robrecht Raedt

**Affiliations:** ^1^4BRAIN, Department of Head and Skin, Ghent University, Ghent, Belgium; ^2^Department of Electrical Engineering, Eindhoven University of Technology, Eindhoven, Netherlands; ^3^Medical Image and Signal Processing, Department of Electronics and Information Systems, Ghent University, Ghent, Belgium

**Keywords:** locus coeruleus (LC), noradrenaline (NA), chemogenetics, GRAB_NE2m_ biosensor, hippocampus (HC), evoked potentials (EPs), epilepsy

## Abstract

**Introduction:**

The locus coeruleus (LC)—noradrenaline (NA) system plays a crucial role in modulating neuronal excitability and plasticity. In epilepsy, the LC-NA system plays an important role in regulating seizure thresholds and severity, with elevated NA release mediating the seizure-suppressing effects of vagus nerve stimulation (VNS). We investigated whether chemogenetic LC activation is able to increase hippocampal NA release and affect hippocampal electrophysiology in anesthetized rats.

**Methods:**

22 male Sprague—Dawley rats were injected with the viral vector AAV9-hSyn-NE2m in the hippocampus to induce expression of the GRAB_NE2m_ biosensor to locally measure changes in extracellular NA. 15/22 rats were injected with the CAV2-PRSx8-hM3Dq hSyn-mCherry viral vector in the LC to express the excitatory DREADD hM3Dq, allowing LC activation with deschloroclozapine (DCZ), and 7/22 with CAV2-PRSx8-GtACR2 as a control. A perforant path stimulation electrode and a dentate gyrus (DG) recording electrode were implanted for local field potential (LFP) and evoked potential (EP) recording as well as a DG optical fiber for GRAB_NE2m_ fluorescence measurement.

**Results:**

In a significant number of rats (7/15) we found an increase in hippocampal NA release, field excitatory post synaptic potential (fEPSP) slope and population spike (PS) amplitude, indicating an increase in excitatory neurotransmission and neuronal output. 4/15 rats showed a decrease in NA release without changes in fEPSP slope or PS amplitude, and 4/15 showed no change in NA release.

**Discussion:**

These findings indicate that chemogenetic activation of the LC-NA system can modulate hippocampal evoked responses, supporting further exploration of its role in health and disease, such as in epilepsy.

## 1 Introduction

The locus coeruleus (LC), a small cluster of noradrenergic neurons in the brainstem, is the primary source of noradrenaline (NA) to the neocortex, cerebellum and hippocampus (Benarroch, 2018). Through its widespread axonal projections, the LC plays a crucial role in various behavioral functions such as attention, arousal, learning and memory (Berridge and Waterhouse, 2003). It does so by modulating excitability and synaptic plasticity of large-scale neuronal networks (Harley, 2007; O’Donnell et al., 2012). Engaging the neuromodulatory function of the LC-NA system could be beneficial in disorders with dysfunctional neuronal circuits. In epilepsy, a neurological disorder characterized by recurrent spontaneous seizures, the LC-NA system is well-known for its anticonvulsant effects (Giorgi et al., 2003). The LC, through input from the nucleus of the solitary tract, has been implicated in the seizure-suppressing effects of vagus nerve stimulation (VNS), with research from our lab and others showing a correlation between VNS and increased hippocampal NA release (Roosevelt et al., 2006; Manta et al., 2009; Raedt et al., 2011; De Taeye et al., 2014; Farrand et al., 2023).

We hypothesize that moderate NA elevation, as seen with VNS, may modulate hippocampal electrophysiology and aim to further explore the LC-NA system as a potential target for drug-resistant epilepsy (DRE). To accomplish our objective, we employed chemogenetic tools, i.e. Designer Receptors Exclusively Activated by Designer Drugs (DREADDs) under control of the noradrenergic PRSx8 promoter, to selectively modulate the LC-NA system. Using the excitatory DREADD hM3Dq, an engineered M3 muscarinic receptor that is unresponsive to its natural ligand acetylcholine but activated by the inert drug deschloroclozapine (DCZ), we aimed to activate LC neurons and consequently elevate hippocampal NA release (Armbruster et al., 2007; Nagai et al., 2020). Chemogenetic modulation of the LC has been widely used to study various brain functions, including memory, anxiety and stress (Fortress et al., 2015; Borodovitsyna et al., 2020; Privitera et al., 2023; Mir et al., 2024). Fortress et al. (2015) demonstrated that chemogenetic activation of the LC, validated through behavioral tests, could prevent memory deficits in a Down Syndrome mouse model. Similarly, Privitera et al. (2023) showed that chemogenetic LC activation replicated stress-induced changes in hippocampal gene expression. Chemogenetic activation of the LC to induce NA release in the hippocampus, combined with an electrophysiological study of hippocampal function, has not previously been explored in the context of targeting the LC-NA system for developing therapies for drug-resistant epilepsy (DRE).

For hippocampal NA measurements, we used a state-of-the-art technique i.e., the GRAB_NE2m_ biosensor (Feng et al., 2019). This GPCR-activation-based (GRAB) NA biosensor enables monitoring of NA release with high temporal resolution (< 1 s), ligand selectivity (94-fold difference in E_max_/EC_50_ between NA and dopamine) and sensitivity (EC_50_ ∼380 nM) (Feng et al., 2024). For the hippocampal electrophysiological investigation, we recorded evoked potentials (EPs) and local field potentials (LFPs) in the dentate gyrus (DG). DG EPs, elicited by electrical stimulation of the perforant path (PP), display a positive field excitatory postsynaptic potential (fEPSP), which reflects synaptic transmission and depolarization of dentate granule cells. When this depolarization reaches a threshold, it generates a secondary superimposed negative peak, known as the population spike (PS), representing the synchronous firing of granule cells. Our study aimed to determine if DCZ-mediated hM3Dq activation in LC noradrenergic neurons could increase hippocampal NA and affect DG electrophysiology in anesthetized rats. Based on prior research, we hypothesized that LC activation would increase hippocampal NA release, leading to a corresponding rise in fEPSP slope, PS amplitude and a reduction in total LFP power (Brown et al., 2005; Harley, 2007; Larsen et al., 2016; Van Lysebettens et al., 2020).

## 2 Material and methods

### 2.1 Animals

Experiments were conducted in male Sprague–Dawley rats (*n* = 22, Envigo, The Netherlands, 300–400 g, 8 weeks old at time of viral vector injection). Rats were housed in a temperature (20–23°C) and humidity (40–60%) controlled room with a fixed 12 h/12 h light/dark cycle and food and water *ad libitum*. All experimental protocols were approved by the Animal Experimental Ethics Committee of Ghent University (ECD 19/42 and 22/54) and complied with the ARRIVE guidelines.

### 2.2 Injection of viral vector

**FIGURE 1:**
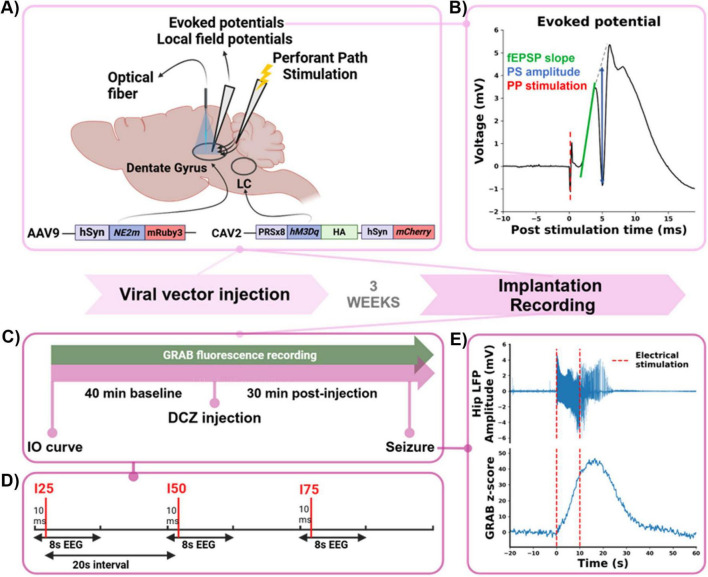
Experimental timeline. **(A)** Schematic overview of bilateral viral vector injection in the LC (PRSx8-hM3Dq-HA hSyn-mCherry, hM3Dq DREADD) and unilateral viral vector injection in the dentate gyrus (DG) of the hippocampus (hSyn-NE2m-mRuby3, GRAB_NE2m_ biosensor). After 3 weeks, an optical fiber was implanted in the hippocampus to perform fiber photometry, an electrode to stimulate the perforant path (PP) and an electrode to record evoked potentials (EPs) and local field potentials (LFPs) in the DG. **(B)** Example of an EP, elicited by PP path stimulation (red), consisting of a positive field excitatory postsynaptic potential (fEPSP, slope indicated in green) and a negative population spike (PS, amplitude indicated in blue). **(C)** Schematic illustration of the recording design. After recording of an IO curve to determine the I25, I50 and I75 stimulation intensities, a 40 min baseline period of recording GRAB_NE2m_ fluorescence and LFP-EP sweeps was followed by a DCZ (0.1 mg/kg, s.c.) injection. After which GRAB_NE2m_, EPs and LFPs were recorded for a 30 min post injection period. Every recording session was finished by electrically evoking a hippocampal seizure. **(D)** Detail of an LFP-EP sweep: 8 s LFP fragments with PP stimulation (red vertical line) at 10 ms using I25, I50, and I75, with a 20 s interstimulus interval. **(E)** Representative example of a hippocampal seizure (LFP in upper graph), evoked by 10 s of electrical stimulation of the PP (red, dashed lines) and the time-locked release of NA (GRAB_NE2m_ z-score, lower graph).

Animals (*n* = 22) were anesthetized with a mixture of isoflurane and medical oxygen (5% induction/2% maintenance) and placed in a robotic stereotactic frame (Neurostar, USA). To induce expression of the GRAB_NE2m_ biosensor in hippocampal neurons, with the neuronal hSyn promoter, glass capillaries and the nano injection robot (flowrate 100 nl/min, Neurostar, USA) were used to inject 500 nl of the AAV9-hSyn-NE2m-mRuby3 viral vector (titer 10^13^ pp./ml, WZ Biosciences, USA) in the dorsal (3.8 AP, 1.9 ML relative to bregma, −3.0 DV from dura) and intermediate (4.5 AP, 3.2 ML relative to bregma, −3.3 DV from dura) hippocampus. Afterward, bregma was lowered 2 mm relative to lambda to avoid the transverse sinus, while targeting the LC (3.9 AP, 1.2 ML relative to lambda). The viral vector CAV2-PRSx8-hM3Dq-HA hSyn-mCherry (titer 0.56×10^12^ pp./ml, IGMM, France) was bilaterally injected at three depths in the LC in 15/22 animals (−5.5, −5.8, −6.1 DV from dura, 600 nl/depth, 100 nl/min) to induce expression of hM3Dq, an excitatory DREADD, for chemogenetic modulation of LC noradrenergic neurons (Figure 1A), (Stevens et al., 2021). This viral vector, which incorporates two promoters, was selected to enable injection localization and spread via staining for the mCherry tag in a pre-study phase. Upon validation of the injection site and successful expression of hM3Dq in the LC, the subsequent experiments were performed with this construct. A control group (*n* = 7/22) was injected with the CAV2-PRSx8-GtACR2-fRed viral vector (titer 0.56×10^12^ pp./ml, IGMM, France), to induce expression of the opsin GtACR2, with the same injection parameters and coordinates as the experimental group. This control group accounts for potential effects associated with viral transduction, transgene expression driven by the PRSx8 promoter in the LC, and the administration of DC. Following each injection, the glass capillary was left in place for 5 min to avoid backflow. After surgery, all animals were subcutaneously injected with Meloxicam (2 mg/kg, Boehringer Ingelheim, Germany) and lidocaine (5% Xylocaine, AstraZeneca, UK) was applied to the wound to minimize pain and discomfort. Animals recovered for at least three weeks, to allow optimal expression of the GRAB_NE2m_ biosensor in the hippocampus and the hM3Dq DREADD in the LC.

### 2.3 Experimental design

#### 2.3.1 Implantation and experiment under anesthesia

Three weeks after viral vector injection, animals (*n* = 22) were anesthetized as described before for stereotactic implantation and acute recordings. An epidural screw electrode was placed in the left frontal bone and serving as a ground/reference electrode for the electrophysiology recording. For acquisition of hippocampal EPs and LFPs, a recording electrode made of polyimide-coated stainless-steel wire (Ø70 μm, California Fine Wire, USA) was implanted in the hilus of the DG (5.3 AP, 3.2 ML relative to bregma).

Furthermore, a custom-made bipolar stimulation electrode, made from two twisted polyimide-coated stainless-steel wires (Ø110 μm, Science products, Germany) was implanted in the right PP (0.0 AP, 4.4 ML relative to lambda). The depth of both the recording and stimulation electrode was adjusted using electrophysiological feedback, to obtain maximal amplitude of EPs in response to 400 μA bipolar square wave pulses (biphasic, 0.2 ms pulse width per phase).

To asses changes in DG GRAB_NE2m_ fluorescence, a Ø200 μm optical fiber (0.54 NA, FT200EMT, Thorlabs, Germany) was implanted in the dorsal hippocampus at the previously injected site (3.8 AP, 1.9 ML relative to bregma, −3.0 DV from dura, Figure 1A). Additional bone screws were placed to fix the electrodes and optical fiber in place with dental cement to the skull.

Following implantation of the electrodes and optical fiber, an EP/LFP recording session under isoflurane anesthesia was performed in every animal (Figure 1C). Input-output (IO) curves were obtained by recording 8 s LFP fragments every 10 s while stimulating the PP with bipolar square wave pulses (0.2 ms pulse width per phase) 10 ms after the start of each LFP fragment to evoke a DG EP. A series of 10 stimulation intensities, increasing incrementally, was repeated five times. For each animal, stimulation intensities corresponding to 25% (I25, range: 25–700 μA), 50% (I50, range: 35–800 μA), and 75% (I75, range: 50–950 μA) of the minimally needed intensity producing the maximum population spike amplitude (PSamp) were identified for subsequent recordings (all stimulation intensities can be found in Supplementary Table 1). During a 40 min baseline period, DG LFP-EP sweeps (8 s LFP fragments with PP stimulation at 10 ms using I25, I50, and I75) were recorded with a 20 s interstimulus interval, combined with simultaneous GRAB_NE2m_ fluorescence measurements. This was followed by a subcutaneous injection of deschloroclozapine (DCZ, 0.1 mg/kg, 3% DMSO in saline, Tocris Bioscience) and a 30 min post-injection recording of LFP-EP sweeps and GRAB_NE2m_ fluorescence (Figure 1D). At the end of the experiment, a hippocampal seizure was evoked with a 10 s tetanic train of electrical square-wave pulses (at the I75 stimulation intensity, range: 50–950 μA, 20 Hz, biphasic, 0.2 ms pulse width per phase, Figure 1E). As evoked seizures induce a profound release of NA in the hippocampus, which was shown in our previous study, we induced a seizure at the end of the experiment to validate our biosensor NA measurement (Larsen et al., 2023).

### 2.4 Electrophysiology and photometry settings

Hippocampal LFPs were high-pass filtered at 1 Hz, amplified 248 times and digitized at 10 kHz with a 16-bit resolution over a ± 10 V input range by an analog-digital converter (NiDAQ card, National Instruments, Austin Texas, USA). A constant-current stimulator (DS4 Bi Phasic Stimulator, Digitimer) was used for electrical stimulation. Custom-written MATLAB software was used for data acquisition and to control stimulation. For recording of GRAB_NE2m_ fluorescence, the PyPhotometry acquisition board was used at the time division setting (subtraction of background illumination) with a sampling rate of 130 Hz (Akam and Walton, 2019). A fluorescence Mini Cube (ilFMC4, Doric, Canada) with an integrated LED was used to emit blue light (470 nm, range: 460–490 nm). The cube features a dichroic mirror to allow the excitation light to pass through while separating the fluorescence emission (∼520 nm, range: 500–550 nm). It also includes narrow bandpass filters to limit the excitation and fluorescence spectra, ensuring precise separation of wavelengths. Additionally, the Mini Cube integrates a photoreceiver that converts incoming photons into an electrical signal for further analysis.

### 2.5 Histology

At the end of the experiment, animals were euthanized with an overdose of sodium pentobarbital (Dolethal, 200 mg/kg, intraperitoneal, Vetoquinol, UK) and transcardially perfused with phosphate buffered saline (PBS) and paraformaldehyde (PFA, 4%, pH 7.4). The brains were isolated from the skull, post-fixed in PFA overnight and cryoprotected for 3–4 days in a 30% sucrose solution. Subsequently the brains were snap-frozen in isopentane and stored in liquid nitrogen. Coronal sections of 40 μm were made using a Cryostat (Leica, Germany) to perform immunohistochemistry. Every 3rd free floating section, containing dorsal hippocampus or LC, was first rinsed in distilled water and incubated in 0.5% and 1% H_2_O_2_, for 30 and 60 min, respectively, to block endogenous peroxidase activity. Following two washing steps of 5 min in PBS, sections were incubated for 45 min in blocking buffer (BB, 0.4% Fish Skin Gelatin and 0.2% Triton X in PBS) to block non-specific antibody binding sites. Slices were then incubated in primary antibody for 1 h at room temperature and overnight at 4°C. Chicken anti-GFP (1:2000, Abcam, UK) was used to visualize GRAB_NE2m_ expression in the hippocampus. Mouse anti-dopamine β hydroxylase (DBH, 1:1000, Merck, Germany) was used to visualize LC neurons and rat anti-HA (1:1000, Merck) to visualize the HA-tag indicating hM3Dq expression. The next day, sections were washed twice in BB for 10 min, followed by 1 h incubation in secondary antibodies: Alexa Fluor 488 goat anti-chicken (1:1000, Abcam, UK), Alexa Fluor 488 or 594 goat anti-mouse (1:1000, Abcam) and Alexa Fluor 488 goat anti-rat (1:1000, Abcam) in darkness. After rinsing twice in PBS for 5 min, a nuclear DAPI staining was performed and after two additional washing steps in PBS, sections were mounted on glass slides and cover slipped with Vectashield H1000 mounting medium (Vector Laboratories, USA). Expression of GRAB_NE2m_ and hM3Dq was qualitatively assessed using epifluorescence microscopy (Nikon, Belgium, 10× magnification). A DAPI filter (excitation: 365/28 nm, emission: 445/50 nm), a FITC filter (excitation: 480/30 nm, emission: 530/40 nm) and a TRITC filter (excitation: 540/25 nm, emission: 620/60 nm) were used to quantify intensity. The level of excitation was kept constant between slices (DAPI: 16%, FITC: 46%, TRITC: 9%). ImageJ was used to perform further analysis. To account for background signal, a region of interest (ROI) corresponding to the background was identified. This ROI was chosen in a region devoid of cells or other fluorescent structures, appearing uniformly dark. The background fluorescence signal from the identified region was subtracted from the fluorescence intensity measurements to correct for nonspecific background noise. Following this background correction, a region of interest (ROI), corresponding to the LC, was determined based on the DBH staining. The level of hM3Dq expression was determined as the mean gray value of the HA immunofluorescence staining in the LC ROI.

### 2.6 Data and statistical analysis

All data was analyzed offline using custom-written MATLAB software (version 2019b, MathWorks, Natick, MA, USA) and Python (version 3.10.13) scripts. For the DG EPs, the fEPSP slope (EPslp) and the population spike amplitude (PSamp) were determined for all three intensities (I25, I50, I75). Using the least-square method, the EPslp was determined by fitting a tangent to the fEPSP between its onset and the start of the PS. The PSamp was calculated as the vertical distance between the peak of the PS and the line connecting the positive peaks before and after the PS (Figure 1B, EP example).

Hippocampal LFP power was calculated by a spectral analysis using the Fast Fourier algorithm. To obtain a frequency resolution of 1 Hz, each 8 s LFP sweep was divided into 1 s windows with a 0.5 s overlap. Total band (1–100 Hz) power of the 1 s segments was obtained by calculating the sum of the power of all frequency bands. LFP power in specific frequency bands was calculated by summing the power within those bands. Before statistical analysis, hippocampal EPs and LFP power data were normalized to the mean of a 10 min baseline period before DCZ injection. GRAB_NE2m_ fluorescence signals recorded with the PyPhotometry system were bandpass filtered using a second-order Butterworth filter (high cutoff: 2 Hz, low cutoff: 0.0001 Hz) with zero-phase filtering via SciPy’s filtfilt function.

To determine significant changes in GRAB_NE2m_ fluorescence, the signal was *z*-scored to a 10 min baseline period before injection of DCZ, by subtracting the mean of the baseline period from the filtered GRAB_NE2m_ signal and dividing it by the standard deviation of the baseline, with the following formula:


$$Z-s\,c\,o\,r\,e=\frac{A\,n\,a\,l\,o\,g_{f\,i\,l\,t}-M\,e\,a\,n_{b\,a\,s\,e\,l\,i\,n\,e}}{S\,T\,D_{b\,a\,s\,e\,l\,i\,n\,e}}$$

The mean z-score was determined for the 5–20 min post-injection period, allowing sufficient time for DCZ to enter the bloodstream and during which the biggest increase in NA and the most pronounced effects on electrophysiology were expected. Animals were categorized based on the GRAB_NE2m_ changes where the mean z-score passed the threshold of 3 or −3 as: “increase” (z-score ≥ 3), “decrease” (z-score ≤ −3) and “no change” (z-score > −3 and < 3). By applying this threshold, the categorization ensures a high degree of confidence in identifying significant changes in NA release and minimizes the likelihood of categorizing minor fluctuations as meaningful changes.

Statistical analyses were conducted using SPSS statistics (version 29.0, IBM corp., Armonk, NY, USA) and graphs were generated by custom-written Python scripts. Outcomes of the different groups (increase, decrease, no change and control) were based on the mean of the 5 to 20 min post-injection period. A one-way ANOVA assessed differences in max GRAB_NE2m_ z-score between groups during a seizure and LFP power. For the EP data, a two-way ANOVA evaluated the effect of factor group, stimulation intensity (I25, I50, I75) and group-by-stimulation intensity interaction. A Spearman rank correlation test was used to determine correlations between changes in GRAB_NE2m_ fluorescence (mean z-score) and changes in EPslp or PSamp. The difference in HA-intensity between groups (increase, decrease, no change group), was evaluated with a one-way ANOVA. Bonferroni correction was applied for *post hoc* testing after ANOVA. Data is represented as mean ± standard error of the mean (SEM), with *n* representing the number of animals per group and statistical significance set at a *p*-value ≤ 0.05.

## 3 Results

### 3.1 Effect of DREADD activation on hippocampal GRAB_NE2m_ fluorescence

**FIGURE 2:**
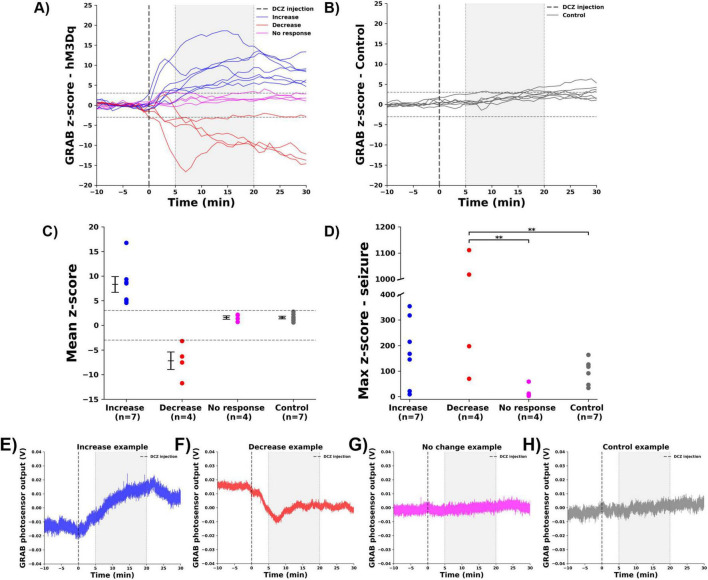
Effect of DCZ injection and seizures on hippocampal GRAB_NE2m_ fluorescence. **(A)** GRAB_NE2m_ fluorescence signal of hM3Dq-expressing rats before and after DCZ injection (dashed vertical line), every line represents one animal. Gray window indicates the selected 5–20 min period to determine the mean z-score. Dashed horizontal lines indicate the significance levels (z-score > 3 or < –3). **(B)** GRAB_NE2m_ fluorescence signal of control rats. **(C)** Mean z-score per group (increase: blue, decrease: red, no change: magenta, control: gray, every dot represents the mean of one animal). **(D)** Max z-score during a seizure for different groups. ** indicates difference between groups with *p* < 0.01. **(E–H)** Representative examples of native GRAB photosensor output in V, reflecting GRAB_NE2m_ fluorescence, for every group [**(E)** increase, **(F)** decrease, **(G)** no change, **(H)** control).

DCZ was administered under anesthesia in hM3Dq-expressing rats (*n* = 15) and control rats (*n* = 7), while continuously monitoring changes in hippocampal NA release with the GRAB_NE2m_ biosensor. DCZ injection resulted in significant changes (*p* < 0.01) in GRAB_NE2m_ fluorescence in 11 out 15 hM3Dq-expressing rats, while no significant changes were observed in the control rats (*p* > 0.01), (Figure 2). hM3Dq-expressing rats were classified in three different groups, based on the mean z-score of GRAB_NE2m_ fluorescence in the 5 to 20 min time window after DCZ injection (gray window, Figures 2A, B). In 7 animals a significant increase (z-score > 3) in GRAB_NE2m_ fluorescence was observed (8.3 ± 1.6, increase group), while 4 animals showed a significant decrease (z-score < −3, −7.2 ± 1.8 decrease group). In 4 other animals, DCZ injection did not significantly change GRAB_NE2m_ fluorescence (1.6 ± 0.3, no change group), (Figure 2C). Figures 2E–H shows representative examples of the native fluorescence traces for each group. Native traces of all animals per group can be found in Supplementary Figure 1.

Evoked seizures are known to cause a profound release of NA in the hippocampus, demonstrated in our previous study (Larsen et al., 2023). Therefore a seizure was induced at the end of the experiment, which consistently increased GRAB_NE2m_ fluorescence in the increase (max z-score: 176.1 ± 50.3), decrease (599.2 ± 270.5), no change (20.3 ± 13.1) and control (100.8 ± 17.6) group, validating the experimental set-up, (Figure 2D). An effect of group was observed (*F* = 5.041, *p* = 0.01), with the decrease group being significantly different (Bonferroni correct) from the no change (*p* = 0.016) and control (*p* = 0.02) group.

### 3.2 Effect of DREADD activation on hippocampal electrophysiology

**FIGURE 3:**
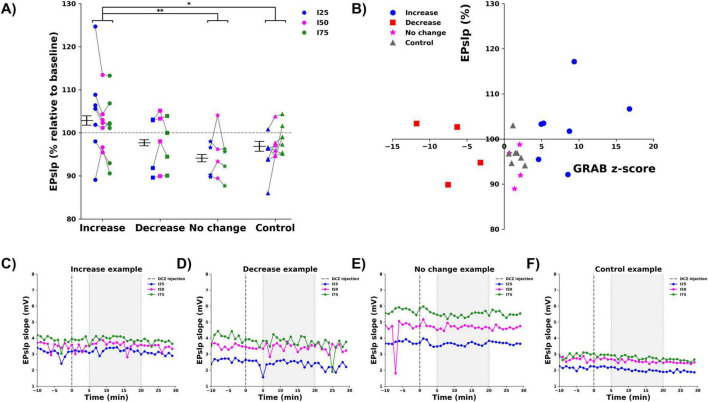
Effect of DCZ injection on the fEPSP slope (EPslp). **(A)** Mean % change relative to baseline per group (increase: 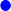, decrease: 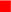, no change: 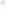, control: 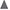), per stimulation intensity (I25: blue, I50: magenta, I75: green). Every dot depicts one animal, gray lines connect data points from one animal. * indicates difference between groups with *p* < 0.05, ** indicates difference between groups with *p* < 0.01. **(B)** No correlation between mean % change in EPslp and mean z-score of GRAB_NE2m_ fluorescence was observed. **(C–F)** Representative examples of the raw EPslp traces before and after DCZ injection of every group [**(C)** increase, **(D)** decrease, **(E)** no change, **(F)** control, I25: blue, I50: magenta, I75: green). Dashed line indicates DCZ injection. Gray window indicates the selected 5–20 min period to determine mean % change.

Dentate gyrus evoked potentials were recorded at three different stimulation intensities (I25, I50, I75) before and after DCZ administration, to evaluate changes in fEPSP slope (EPslp), a marker of synaptic strength, in PS amplitude (PSamp), a marker for postsynaptic activation of DG neurons. DCZ injection resulted in a significant change in both the EPslp and PSamp, in the 5 to 20 min period after injection. For the EPslp, (Figure 3A), there was a significant effect of group factor (*F* = 5.796, *p* = 0.002), with the EPslp of the increase group (102.9 ± 1.1%) being significantly higher than the no change (94.1 ± 0.8%, *p* = 0.002) and the control group (96.9 ± 1.2%, *p* = 0.020), Bonferroni corrected. No significant effect of the stimulation intensity (*F* = 0.165, *p* = 0.848) or the interaction between group and stimulation intensity (*F* = 0.534, *p* = 0.78) was found. To assess correlation between the observed changes in GRAB_NE2m_ fluorescence and the EPslp, a Spearman rank correlation test was performed, showing no correlation between the two parameters (Spearman’s rho correlation = 0.290, *p* = 0.191, two-tailed), (Figure 3B). Figures 3C–F shows representative examples of the raw changes in fEPSP slope from one animal in each group. Data of all animals per group can be found in Supplementary Figure 2.

**FIGURE 4:**
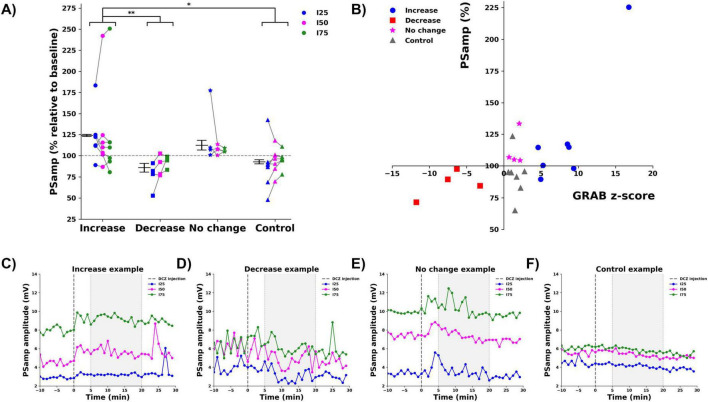
Effect of DCZ injection on the PS amplitude (PSamp). **(A)** Mean % change relative to baseline per group (increase: 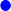, decrease: 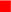, no change: 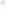, control: 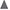), per stimulation intensity (I25: blue, I50: magenta, I75: green). Every dot depicts one animal, gray lines connect data points from one animal. * indicates difference between groups with *p* < 0.05, ** indicates difference between groups with *p* < 0.01. **(B)** A significant correlation between mean % change in PSamp and mean z-score of GRAB_NE2m_ fluorescence was observed. **(C–F)** Representative examples of the raw PSamp traces before and after DCZ injection of every group [**(C)** increase, **(D)**: decrease, **(E)** no change, **(F)** control, I25: blue, I50: magenta, I75: green). Dashed line indicates DCZ injection. Gray window indicates the selected 5–20 min period to determine mean % change.

For the PSamp, (Figure 4A), a main effect was found for group (*F* = 5.056, *p* = 0.004). Pairwise comparisons (Bonferroni corrected) indicated a significant increase in PSamp for the increase group (123.9 ± 1.2%) compared to both the decrease (85.7 ± 5.2%, *p* = 0.011) and the control (92.6 ± 2.4%, *p* = 0.016) groups. No significant effect of the stimulation intensity (*F* = 0.24, *p* = 0.976) or interaction between group and stimulation intensity (*F* = 0.265, *p* = 0.951) was found. A Spearman rank correlation analysis revealed that the changes in PSamp were significantly correlated to the observed changes in GRAB_NE2m_ fluorescence (Spearman’s rho correlation = 0.476, *p* = 0.025, two-tailed), (Figure 4B). Figures 4C–F shows representative examples of the raw changes in PS amplitude from one animal in each group. Data of all animals per group can be found in Supplementary Figure 3.

**FIGURE 5:**
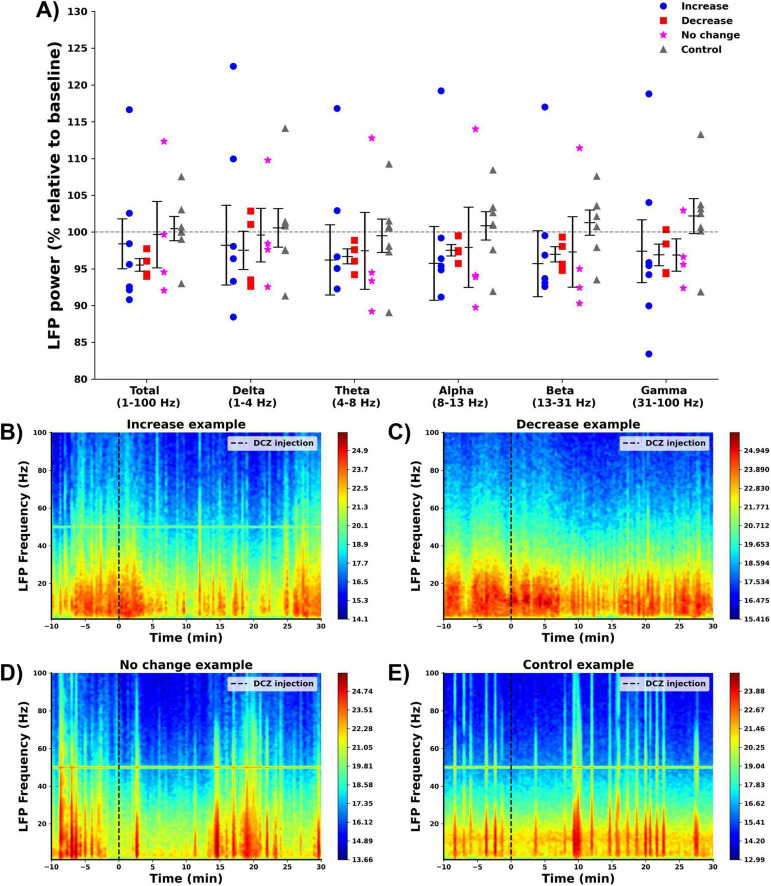
Effect of DCZ injection on hippocampal LFPs. **(A)** Mean % change relative to baseline in total LFP power and LFP power per frequency band per group (increase: 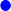, decrease: 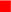, no change: 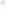, control: 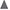). No significant effects were found between the different groups. **(B–E)** Representative examples of the power spectrum of the hippocampal LFP before and after DCZ injection of every group [**(B)** increase, **(C)** decrease, **(D)** no change, **(E)** control).

A power spectral analysis was performed on the hippocampal LFPs recorded before and after DCZ injection, to obtain total band power (1–100 Hz) and the power in different frequency bands (Figure 5). The effect of group on total LFP power was not statistically significant (*F* = 0.465, *p* = 0.710) and no significant differences were found when looking at the different frequency bands: delta (*F* = 0.107, *p* = 0.955), theta (*F* = 0.167, *p* = 0.917), alpha (*F* = 0.353, *p* = 0.787), beta (*F* = 0.569, *p* = 0.642) and gamma band (*F* = 0.675, *p* = 0.579). Figures 5B–E shows representative examples of the LFP power spectrum from one animal in each group.

### 3.3 Histological analysis of DREADD and GRAB_NE2m_ expression

**FIGURE 6:**
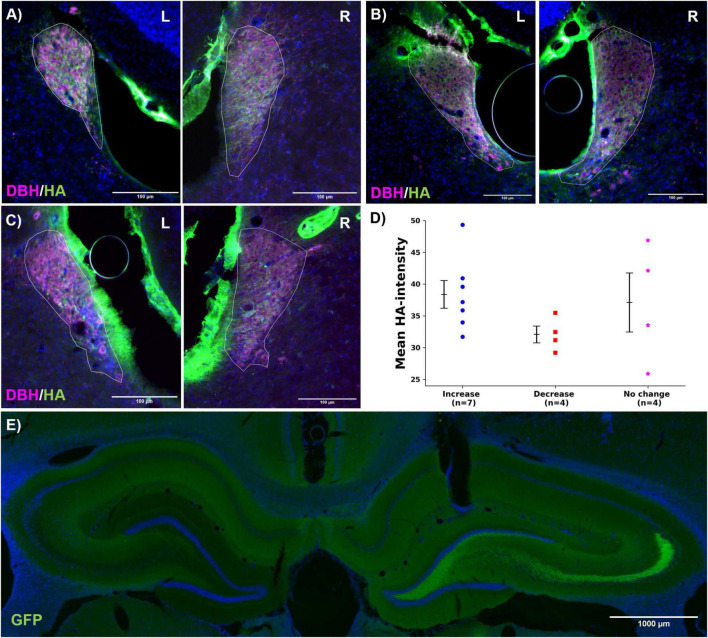
Histology. **(A–C)** Representative images of hM3Dq expression (HA, green) in the left and right LC (DBH, magenta, LC ROI indicated with white line) for the increase, decrease and no change group, respectively. Overlap between the dopamine-beta-hydroxylase (DBH, magenta) and hM3Dq-positive neurons (HA, green) indicates LC-specific expression. The scale bar measures 100 μm. **(D)** Mean HA-intensity per group (increase: 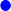, decrease: 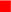, no change: *). Every dots represents one animal. No significant effect was found between the different groups. **(E)** Representative image of GRAB_NE2m_ expression in the hippocampus (GFP, green). The scale bar measures 1,000 μm.

In all hM3Dq-expressing rats (*n* = 15), a consistent DREADD expression pattern was observed. Figures 6A–C presents representative images for each group, detailed images can be found in Supplementary Figure 4. Expression levels were evaluated using epifluorescence microscopy, with the mean intensity of HA immunofluorescence in the LC (ROI) serving as the outcome parameter. DREADD expression was confined to DBH-positive neurons in all cases, confirming LC specific expression. Some autofluorescence was observed outside the LC, likely due to the use of a rat anti-HA antibody, which stained nearby vessels. No significant difference in HA intensity among the three groups could be observed (mean gray values: increase, 38.4 ± 2.2; decrease, 32.1 ± 1.3; no change, 37.1 ± 4.6; *F* = 1.300, *p* = 0.308, Figure 6D). GRAB_NE2m_ expression was detected in the dentate gyrus of the hippocampus in all animals (Figure 6E). Detailed images of the optical fiber and recording electrode location can be found in Supplementary Figure 5.

## 4 Discussion

In this study, we found that chemogenetic LC-activation induced significant changes in both hippocampal NA release and electrophysiology. In accordance with our hypothesis, we found an increase in hippocampal NA release in 7 out of 15 hM3Dq-expressing rats. In these rats there was a corresponding significant increase in fEPSP slope and PS amplitude, reflecting increased glutamatergic neurotransmission and neuronal activation.

Our findings are in line with earlier pharmacological studies demonstrating that NA is able to potentiate the amplitude of the PS in DG EPs, induced by PP stimulation, and enhances excitability of DG granule cells (Richter-Levin et al., 1991; Knight and Harley, 2006; Harley, 2007). The effect of increased NA release on the fEPSP slope has been more variable. Pharmacological *in vitro* studies have shown an increase in the fEPSP slope, similar to what we observed in our study, but consistent effects have been absent in *in vivo* studies (Harley and Milway, 1986; Lacaille and Harley, 1985). Despite these inconsistencies, several studies involving VNS have reported a significant VNS-induced increase in the fEPSP slope, which is attributed to a NA-induced increase in glutamate release (Shen et al., 2012; Ura et al., 2013; Van Lysebettens et al., 2020). Enhancing effects of NA on fEPSP slope and PS amplitude are mainly mediated through activation of the β-adrenergic receptor (β-AR) in DG granule cells (Munro et al., 2001; Harley, 2007). Through β-AR activation, NA boosts the cAMP-PKA signaling cascade in DG granule cells, leading to enhanced glutamate release and increased dentate granule cell response to given inputs (Harley, 2007; Hagena et al., 2016). In animals with decreased or no change in NA release, there were no observable changes in the fEPSP slope or PS amplitude compared to the control group, likely explained by the low affinity of β-adrenergic receptors, requiring higher NA concentrations for activation and downstream effects (Ramos and Arnsten, 2007). Consistent with this view, Bouret and Sara (2005) and Sara and Bouret (2012) described the so-called network reset theory, where LC activation, through β-adrenergic receptor signaling, enhances stimulus-evoked activity and suppresses irrelevant activity, enabling shifts in attention, cognition and behavior to prioritize survival-relevant information. Additionally, suppressing irrelevant baseline activity might be a key mechanism in reducing the likelihood of epileptic seizures by preventing the spread of abnormal neuronal firing patterns. Future studies will need to investigate the impact of LC activation on electrophysiology in a chronically hyperexcitable hippocampus of epileptic rats.

Although population spikes are primarily generated by the synchronized firing of granule cells (Andersen et al., 1971; Bragin et al., 1995), the dentate gyrus microcircuit involves multiple interacting cell types that modulate granule cell activity (Amaral et al., 2007). Hilar mossy cells, which receive significant noradrenergic input, provide excitatory feedback to granule cells and activate inhibitory interneurons, creating a complex regulatory loop (Scharfman, 1995; Lacefield et al., 2012; Jinde et al., 2013; Scharfman and Myers, 2013). Similarly, somatostatin-positive interneurons, which inhibit granule cell dendrites, are sensitive to NA and may modulate dendritic input integration (Tallent, 2007; Lovett-Barron et al., 2012; Fanselow et al., 2016; Yuan et al., 2017). Astrocytes, through noradrenaline-dependent gliotransmitter release and ionic regulation, further influence granule cell excitability and synchrony (Perea et al., 2009; Wahis and Holt, 2021). These indirect mechanisms highlight the importance of considering the entire microcircuit when interpreting NA-induced changes in hippocampal electrophysiology, as observed in this study.

In the rats showing a NA increase, DCZ-induced hM3Dq activation resulted in a mean rise of 8.31 z-scores from baseline, corresponding to a moderate elevation of NA release. The LC typically fires in a tonic activation mode, with the highest discharge rates during waking (> 2 Hz during active waking) and lower rates during sleep (< 1 Hz) (Berridge and Waterhouse, 2003). This baseline activity maintains steady NA release, which supports normal attention and arousal levels. In response to sensory stimuli, LC neurons exhibit phasic bursts of activity, leading to transient increases in NA release that enhance focus and facilitate rapid responses to salient stimuli (Berridge and Waterhouse, 2003). Importantly, NA release is closely tied to LC firing rates, with even small fluctuations in LC activity producing significant changes in NA release (Berridge and Abercrombie, 1999). DCZ-induced hM3Dq activation stimulates Gq signaling, depolarizing neuronal resting membrane potentials and increasing tonic LC firing (Oyarzabal et al., 2022). This activation elevates NA release in LC projection areas, including the hippocampus and DG, the latter receiving particularly dense NA innervations (Blackstad et al., 1967; Verhage et al., 1992). Our results support this mechanism, showing that hM3Dq-induced LC activation enhances tonic firing, leading to an increase in hippocampal NA. Seizure-induced activity, however, led to a much larger NA increase (mean max *z*-score of 176) compared to chemogenetic activation (mean increase of 8.31 z-scores).

This is consistent with our earlier findings, which demonstrated a high increase in LC firing and the time-locked release of NA during electrically evoked seizures in the hippocampus (Larsen et al., 2023). Together, these findings suggest that chemogenetic activation of the LC induces a more moderate rise in tonic LC firing, resulting in a correspondingly moderate increase in NA release. In contrast, seizures are associated with a pronounced increase in LC firing, leading to a substantial elevation in NA release. These observations highlight the finely regulated nature of the LC-NA system, which modulates NA levels in response to varying degrees of LC activation (Berridge and Abercrombie, 1999).

In a subset of rats, we observed either a decrease (4 out of 15) or no change (4 out of 15) in hippocampal NA release. Auto-inhibitory mechanisms in the hippocampus are one possible explanation of why some animals showed no change or a decrease in NA release. NA released either locally (in the LC) or in projection areas, such as the hippocampus binds to presynaptic α_2_-adrenergic (AR) auto-receptors located on noradrenergic nerve terminals. Activation of these receptors triggers an auto-inhibition mechanism, inhibiting further release of NA from the same or nearby terminals to ensure a steady baseline level of NA in the brain (Aghajanian et al., 1977). In one “decrease” and one “no change” animal, a first small increase in hippocampal NA followed by a moderate or strong decrease was visible, suggesting the activation of this auto-inhibition loop in the hippocampus. In most decrease animals, however, an immediate decrease in NA was observed, which might be explained by higher local release of NA in the LC due to high activation of the LC, triggering autoinhibition of further NA release along the axonal terminals to the projection areas. This auto-inhibitory mechanism could be further explored with α_2_-AR antagonists, however, given the use of an α_2_-AR based GRAB_NE2m_ biosensor, this was not possible in this study. Future experiments utilizing the recently developed nLight biosensor, which is based on α_1_-AR, may provide valuable insights into the auto-inhibition-mediated reduction in NA release following chemogenetic activation of the LC (Kagiampaki et al., 2023).

In all animals, an increase of NA release was observed during electrically evoked hippocampal seizures. This release was very high compared to the moderate hM3Dq-induced release of NA observed in 7/15 animals. This infers that no auto-inhibition mechanisms are at play that prevent this release from happening. As shown in our previous study, hippocampal seizures are associated with the increase in firing of a subset of LC neurons. The corresponding massive release of NA is therefore attributed to the strong excitatory drive that the LC receives during a seizure from the amygdala and the prefrontal cortex (Breton-Provencher et al., 2021). Additionally, we propose that strong hippocampal activation during seizures generates potent extracellular potential fields, which activate voltage-gated ion channels in hippocampal-projecting LC axons, resulting in high local NA release. We hypothesize that these intense excitatory inputs override auto-inhibition mechanisms, thereby preventing their regulatory effects on the massive release of NA.

Histological analysis confirmed hM3Dq expression in the LC of all animals. However, a detailed retrograde labeling study to specifically assess DREADD expression in hippocampus-projecting LC neurons was not conducted, which is a limitation of this study. As a result, variations in expression patterns across individual animals are likely. In a study of Vazey and Aston-Jones (2014) successful hM3Dq expression in LC neurons resulted in increased firing rates in most rats, yet in a subset, LC activation paradoxically led to a decrease in firing rate. It was hypothesized that these neurons, possibly with lower hM3Dq expression, were inhibited by local NA release from nearby hM3Dq-expressing, activated neurons (Vazey and Aston-Jones, 2014). Also Hirschberg et al. (2017) and Davy et al. (2022) demonstrated that activation of the excitatory chemogenetic receptor PSAM reduced LC firing rates or intracellular calcium levels in certain LC neurons. Collectively, these studies suggest that the observed decrease in hippocampal NA release may be explained by lateral inhibition within the LC due to variations in DREADD expression patterns (Davy et al., 2022). As a retrograde labeling study was not conducted, it is improbable that variations in expression could be discerned from the experimental data presented in this study. This is recognized as a limitation of our study.

The hippocampus primarily receives input from the dorsal part of the LC (Waterhouse et al., 1983). In animals with increased hippocampal NA release, the dorsal LC may therefore have been preferentially activated by hM3Dq. To determine whether LC neurons projecting to the hippocampus are more activated in animals exhibiting increased hippocampal NA release, a retrograde labeling study similar to that conducted by Borodovitsyna et al. (2020), would be needed. This would involve injecting a CAV viral vector into the hippocampus to induce hM3Dq expression in hippocampus-projecting LC neurons, combined with measures of LC activity such as unit recordings or GCaMP signaling.

Anesthesia is known to induce hyperpolarization of neurons, including LC neurons, through an increase in K^+^ conductance (Sirois et al., 2000). In our previous paper, however, we demonstrated that LC neurons are firing tonically even under isoflurane anesthesia (Larsen et al., 2023). In this study, we did not conduct unit recordings to confirm hM3Dq-induced activation of the LC. Consequently, variations in anesthesia depth or the extent of hyperpolarization of LC neurons was not recorded and may have led to insufficient hM3Dq-induced depolarization. This could result in the action potential firing threshold not being reached, preventing downstream release of NA in animals that exhibited no change or a decrease in NA levels. Additionally it is known that hyperpolarization-activated cyclic nucleotide-gated (HNC) channels, amongst other voltage-gated ion channels, play a crucial role in the tonic firing of LC neurons (Hasan et al., 2022). When the membrane potential becomes more negative (hyperpolarized), these channels open and allow cations (such as Na^+^ and K^+^) to flow in, which leads to a depolarizing current. This current helps to bring the membrane potential back toward a threshold after each action potential, contributing to a constant low level of depolarization that underlies tonic firing (Robinson and Siegelbaum, 2003). hM3Dq inhibits KCNQ channels and induces the release of calcium from internal stores, resulting in a slight depolarization (Alexander et al., 2009). This depolarization can inactivate HCN channels thereby reducing the depolarizing current. A diminished depolarizing current might disrupt the balance between depolarizing and hyperpolarizing forces, impairing the neuron’s ability to maintain rhythmic firing and which might reduce LC tonic firing and downstream NA release. Taken together, the influence of anesthesia, combined with variability in hM3Dq activation strength and its effects on ion channel activity, could explain the observed differences in NA levels. Specifically, insufficient or excessive depolarization could either inhibit ion channels critical for tonic firing or fail to reach the threshold for action potential firing, ultimately leading to no or reduced release of NA.

In contrast to our hypothesis, where we expected a decrease in total LFP power, no significant changes were observed in animals with elevated hippocampal NA release. The effects of NA release on LFPs are known to be variable, with Brown et al. (2005), showing that glutamate-induced LC activation in urethane-anesthetized rats increased DG theta activity in the 4–8 Hz range, while Walling et al. (2011) observed this only in awake rats and was unable to replicate it under anesthesia. These findings suggest that the lack of significant effects of NA on spontaneous LFPs observed in this study might be explained by the anesthetized state of the animal. During anesthesia, hippocampal LFP patterns show characteristics that reflect neural suppression. Isoflurane, a volatile anesthetic, shifts LFP activity toward low-frequency, high-amplitude waves, primarily in the delta and theta ranges (Ferron et al., 2009). Unlike urethane anesthesia, isoflurane heavily obscures the brain’s naturally occurring functional connectivity, signifying a deep and stable state of unconsciousness accompanied by a marked reduction in cortical and subcortical excitability (Ferron et al., 2009; Paasonen et al., 2018). This suppression likely explains the lack of significant effect of NA release on LFPs observed in this study.

Conducting experiments under anesthesia is recognized as a limitation of this study. Nevertheless, the results demonstrate that NA release, even under anesthetized conditions, induces significant effects on hippocampal electrophysiology, providing critical insights into the mechanistic role of NA in modulating neural activity. Moreover, the findings highlight the importance of employing a biosensor to accurately monitor and control the outcomes of LC modulation studies. These findings lay a foundation for future studies in awake, freely behaving animals to further elucidate the behavioral and physiological relevance of LC-mediated neuromodulation.

## 5 Conclusion

This study uniquely combines chemogenetic LC modulation, GRAB_NE2m_ biosensor technology and hippocampal electrophysiology to investigate NA release in relation to hippocampal excitability. We demonstrated that a DCZ injection in hM3Dq-expressing rats is able to induce significant changes in hippocampal NA release, resulting in the modulation of hippocampal neurotransmission and neuronal output. The achieved modulation of the LC-NA system supports further exploration of its role in health and disease, such as in chronic epilepsy models.

## Data Availability

The original contributions presented in this study are included in this article/Supplementary material, further inquiries can be directed to the corresponding author.

## Supplementary material

Supplementary Figure 1 Native GRABne fluorescence traces per group. **(A–D)** Native GRAB photosensor output traces (in V), reflecting GRAB_NE2m_ fluorescence, for every animal (depicted by one line/color) per group [**(A)** increase, **(B)** decrease, **(C)**: no change, **(D)**: control).

Supplementary Figure 2 Raw fEPSP slope traces per group. **(A–C)** EPslp trace (in mV) of every animal in the increase group for every stimulation intensity (I25, I50, I75). **(D–F)** EPslp trace for the decrease group. **(G–I)** EPslp trace for the no change group. **(J–L)** EPslp trace for the control group.

Supplementary Figure 3 Raw PSamp traces per group. **(A–C)** PSamp trace (in mV) of every animal in the increase group for every stimulation intensity (I25, I50, I75). **(D–F)** PSamp trace for the decrease group. **(G–I)** PSamp trace for the no change group. **(J–L)** PSamp trace for the control group.

Supplementary Figure 4 DREADD expression in the LC. Detailed representative images of hM3Dq expression (HA, green) in the left and right LC (DBH, magenta), overlap between hM3Dq and DBH in the third panel shows LC specific expression. The scale bar measures 100 μm. **(A)** Representative images of the increase group. **(B)** Representative images of the decrease group. **(C)** Representative images of the no change group.

Supplementary Figure 5 Fiber and electrode placement in the hippocampus. **(A)** Location of the optical fiber. **(B)** Location of the recording electrode in the dentate gyrus. The scale bar in all images measures 500 μm.
