# Supplementary material for: Chemogenetic modulation of the rat locus coeruleus alters hippocampal noradrenaline release and modulates perforant path-evoked responses
Source: Front Neurosci. 2025 Feb 19;19:1544830. doi: 10.3389/fnins.2025.1544830 (PMC11880610; doi:10.3389/fnins.2025.1544830)
Supplement: Supplementary file 1 [file Table_1.DOCX]

**Supplementary table 1**. Intensity values (in µA) for the I25, I50, I75 and tetanic train stimulation, for every animal.

| - **Group and animal ID** | - **I25 intensity (µA)** | - **I50 intensity (µA)** | - **I75 and tetanic train intensity (µA)** |
| --- | --- | --- | --- |
| - Increase 01 | - 500 | - 700 | - 950 |
| - Increase 02 | - 600 | - 800 | - 950 |
| - Increase 03 | - 700 | - 800 | - 950 |
| - Increase 04 | - 300 | - 400 | - 500 |
| - Increase 05 | - 65 | - 80 | - 100 |
| - Increase 06 | - 190 | - 250 | - 300 |
| - Increase 07 | - 25 | - 35 | - 50 |
| - Decrease 01 | - 300 | - 500 | - 800 |
| - Decrease 02 | - 100 | - 130 | - 150 |
| - Decrease 03 | - 50 | - 100 | - 150 |
| - Decrease 04 | - 125 | - 200 | - 250 |
| - No change 01 | - 600 | - 700 | - 950 |
| - No change 02 | - 500 | - 700 | - 800 |
| - No change 03 | - 350 | - 500 | - 700 |
| - No change 04 | - 300 | - 500 | - 800 |
| - Control 01 | - 200 | - 350 | - 500 |
| - Control 02 | - 100 | - 200 | - 400 |
| - Control 03 | - 300 | - 400 | - 500 |
| - Control 04 | - 200 | - 500 | - 700 |
| - Control 05 | - 200 | - 400 | - 600 |
| - Control 06 | - 100 | - 200 | - 500 |
| - Control 07 | - 400 | - 500 | - 700 |
