# Supplementary figures and images for: Chemogenetic modulation of the rat locus coeruleus alters hippocampal noradrenaline release and modulates perforant path-evoked responses
Source: Front Neurosci. 2025 Feb 19;19:1544830. doi: 10.3389/fnins.2025.1544830 (PMC11880610; doi:10.3389/fnins.2025.1544830)

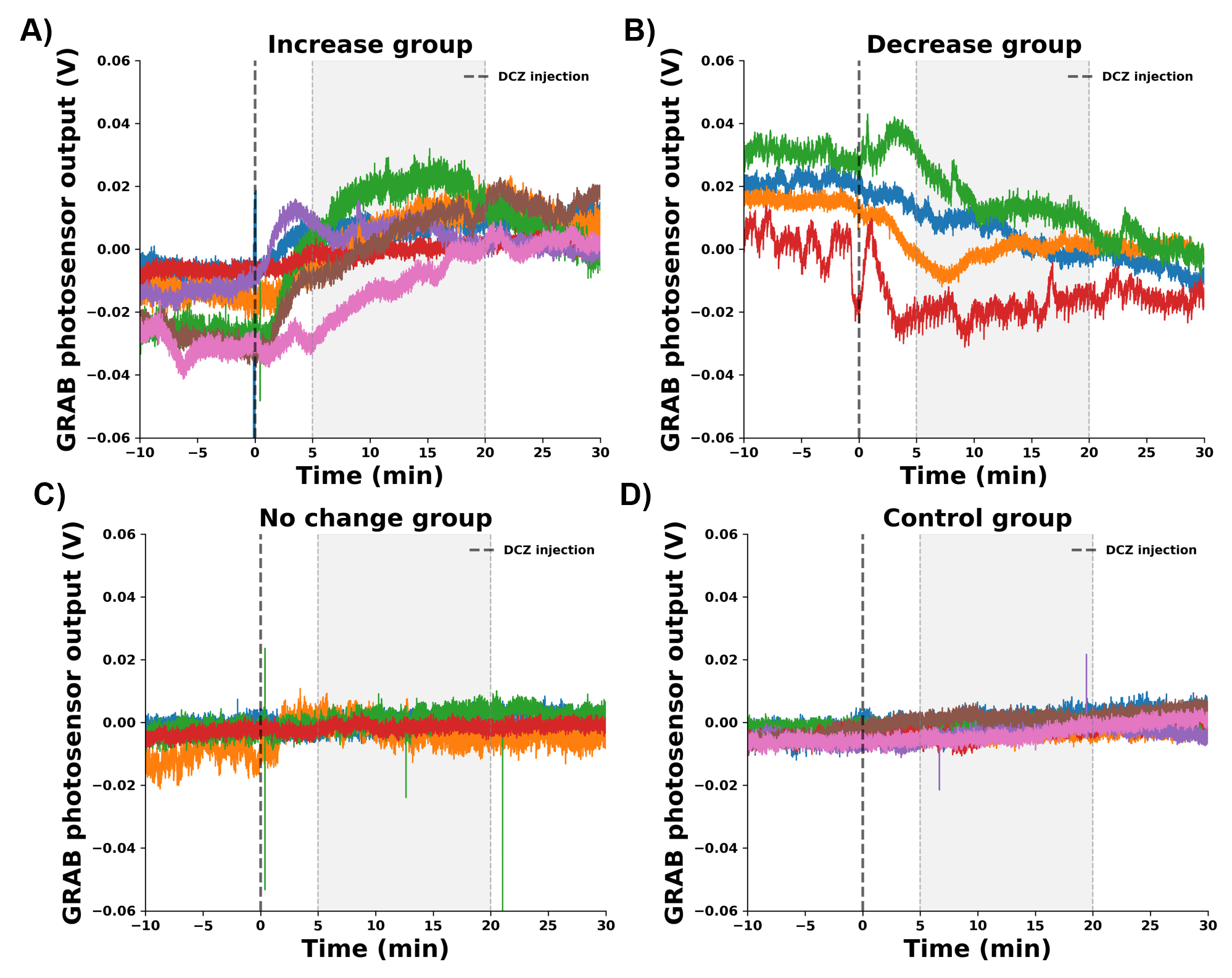

Supplement: Supplementary Figure 1 — Native GRABne fluorescence traces per group. (A–D) Native GRAB photosensor output traces (in V), reflecting GRABNE2m fluorescence, for every animal (depicted by one line/color) per group [(A) increase, (B) decrease, (C): no change, (D): control). [file Image_1.tif]

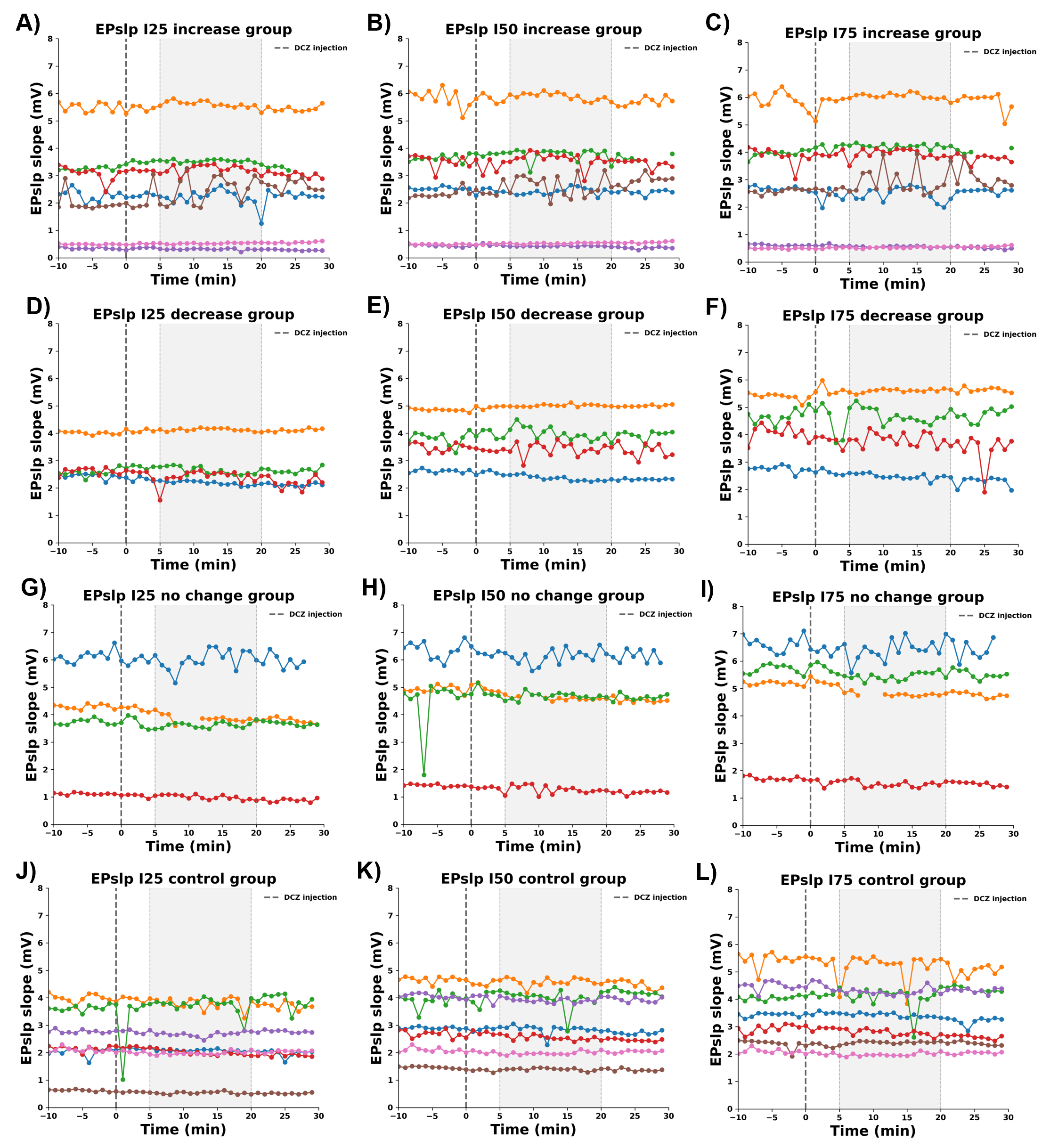

Supplement: Supplementary Figure 2 — Raw fEPSP slope traces per group. (A–C) EPslp trace (in mV) of every animal in the increase group for every stimulation intensity (I25, I50, I75). (D–F) EPslp trace for the decrease group. (G–I) EPslp trace for the no change group. (J–L) EPslp trace for the control group. [file Image_2.tif]

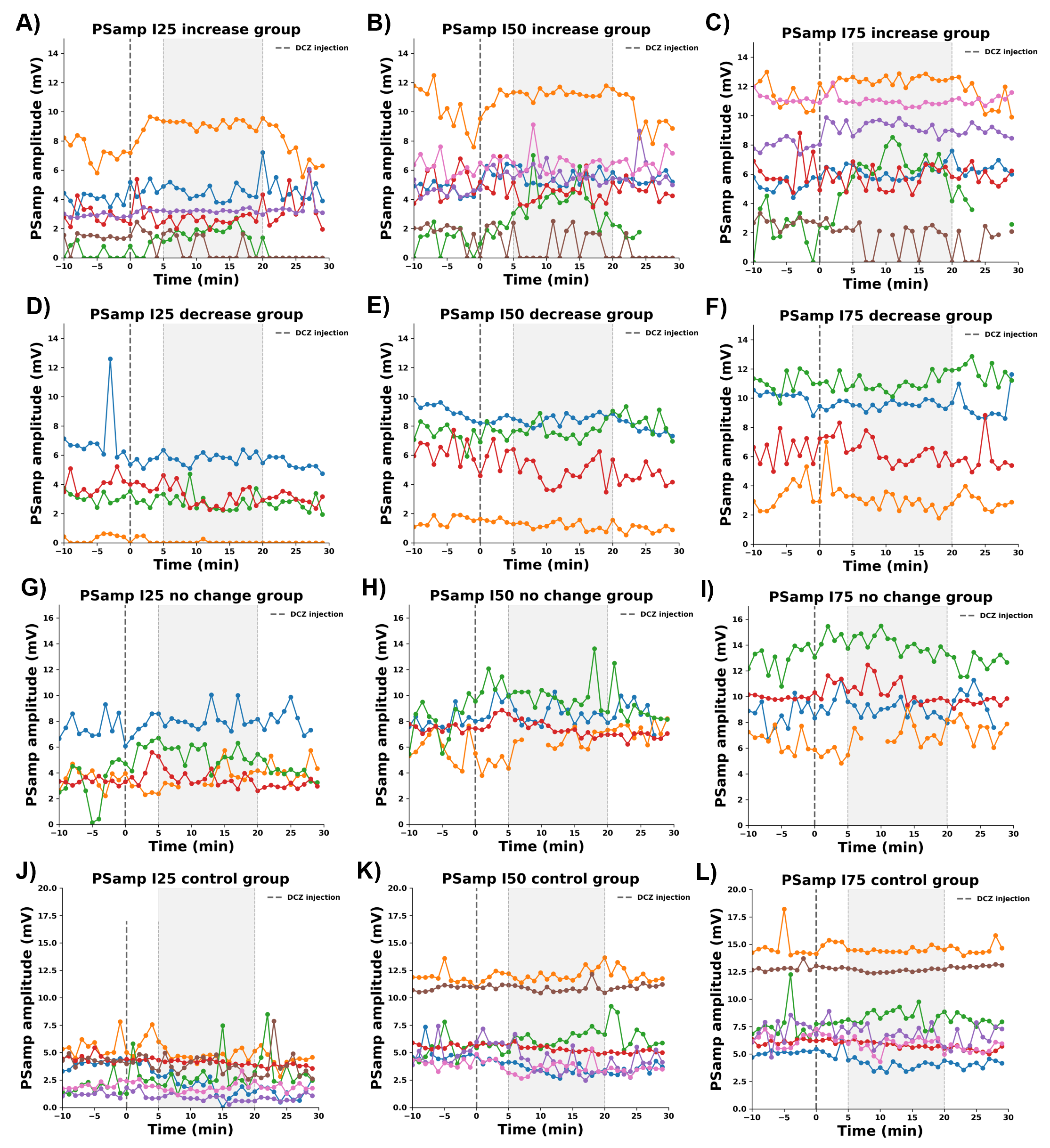

Supplement: Supplementary Figure 3 — Raw PSamp traces per group. (A–C) PSamp trace (in mV) of every animal in the increase group for every stimulation intensity (I25, I50, I75). (D–F) PSamp trace for the decrease group. (G–I) PSamp trace for the no change group. (J–L) PSamp trace for the control group. [file Image_3.tif]

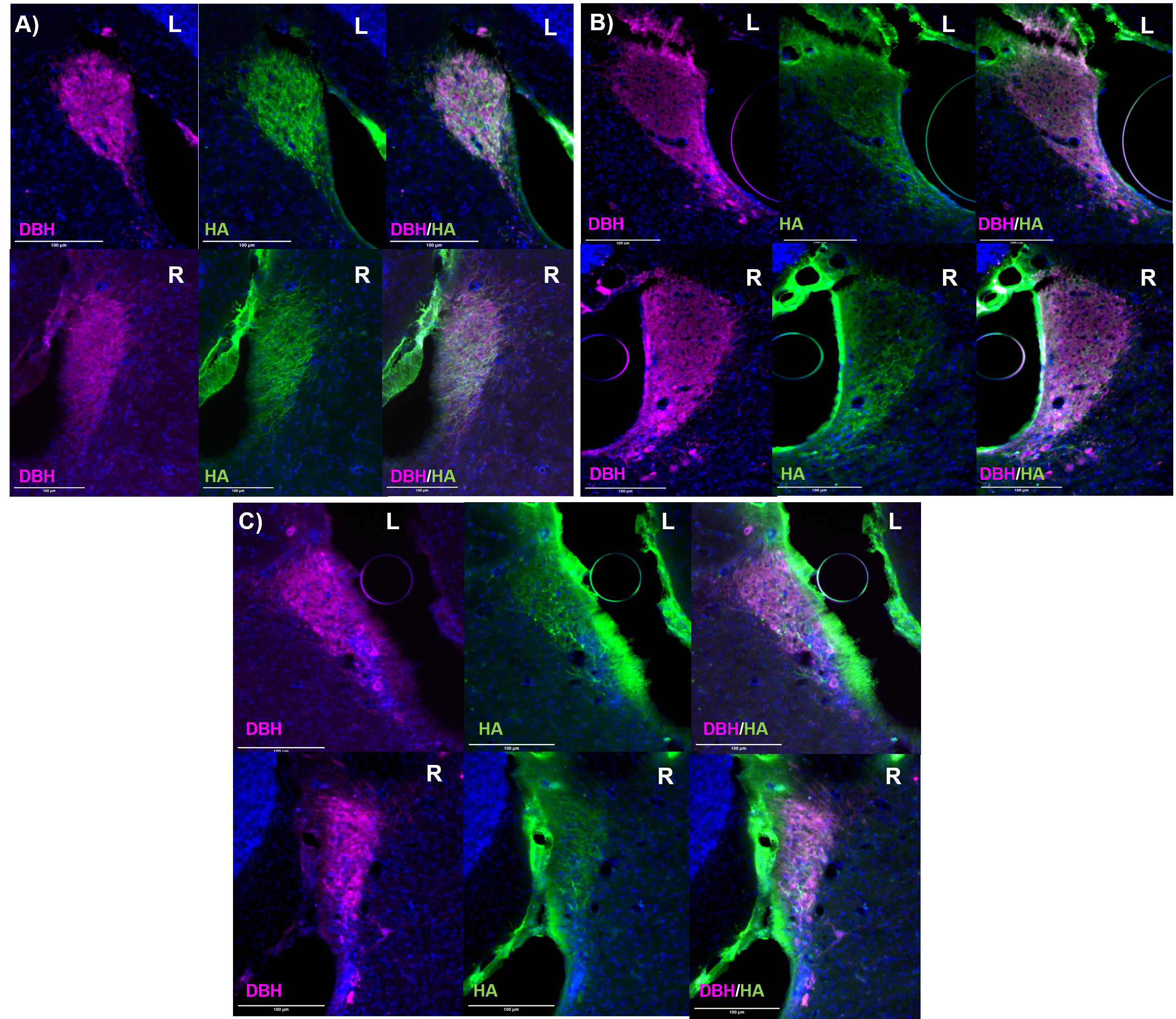

Supplement: Supplementary Figure 4 — DREADD expression in the LC. Detailed representative images of hM3Dq expression (HA, green) in the left and right LC (DBH, magenta), overlap between hM3Dq and DBH in the third panel shows LC specific expression. The scale bar measures 100 μm. (A) Representative images of the increase group. (B) Representative images of the decrease group. (C) Representative images of the no change group. [file Image_4.tif]

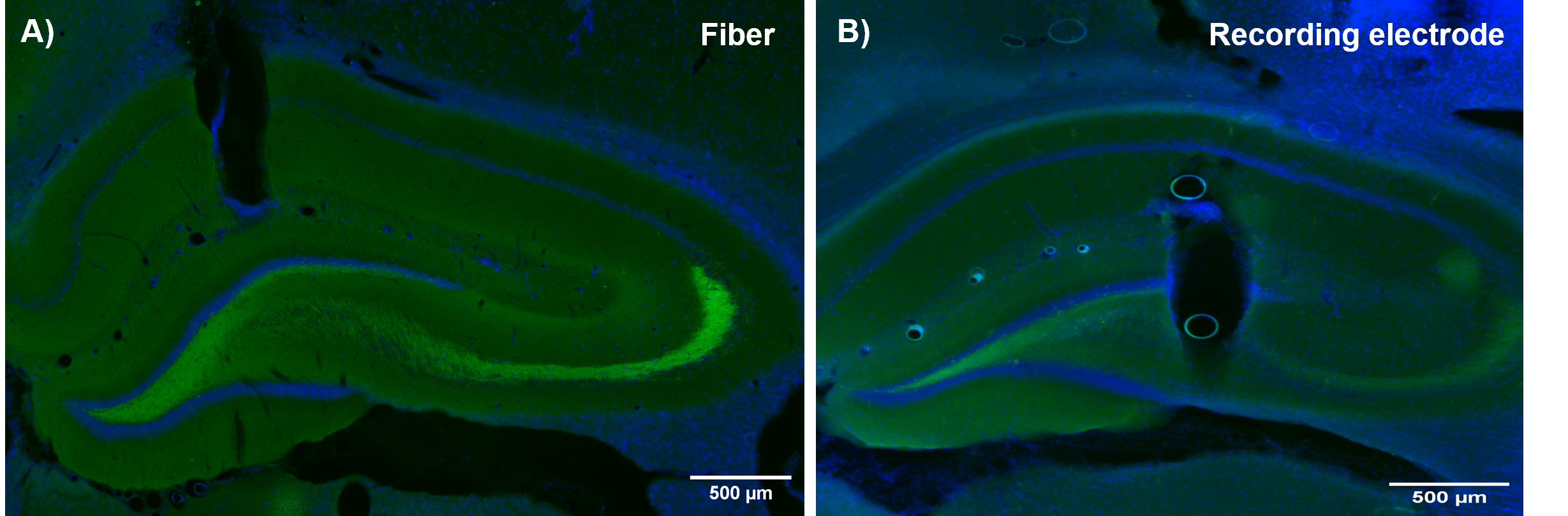

Supplement: Supplementary Figure 5 — Fiber and electrode placement in the hippocampus. (A) Location of the optical fiber. (B) Location of the recording electrode in the dentate gyrus. The scale bar in all images measures 500 μm. [file Image_5.tif]
